# High-efficient Synthesis of Graphene Oxide Based on Improved Hummers Method

**DOI:** 10.1038/srep36143

**Published:** 2016-11-03

**Authors:** Huitao Yu, Bangwen Zhang, Chaoke Bulin, Ruihong Li, Ruiguang Xing

**Affiliations:** 1 College of Materials and Metallurgy, Inner Mongolia University of Science and Technology, Baotou, 014010, P. R. China

## Abstract

As an important precursor and derivate of graphene, graphene oxide (GO) has received wide attention in recent years. However, the synthesis of GO in an economical and efficient way remains a great challenge. Here we reported an improved NaNO_3_-free Hummers method by partly replacing KMnO_4_ with K_2_FeO_4_ and controlling the amount of concentrated sulfuric acid. As compared to the existing NaNO_3_-free Hummers methods, this improved routine greatly reduces the reactant consumption while keeps a high yield. The obtained GO was characterized by various techniques, and its derived graphene aerogel was demonstrated as high-performance supercapacitor electrodes. This improved synthesis shows good prospects for scalable production and applications of GO and its derivatives.

Graphene^1^, a single layer of carbon atoms bonded into a honeycomb two-dimensional lattice, has attracted great interests from scientists and engineers because of its extraordinary properties and wide applications^2^,^3^, such as functional films, electric devices and energy storage devices (Li-ion batteries, supercapacitors) etc. Different from graphene, which is almost not soluble and cannot be dispersed in water or any organic solvent^3^,^4^, graphene oxide (GO) contains high-density oxygen functional groups, like hydroxyl and epoxy group on its basal plane, and carboxyl at its edge^4^,^5^. They afford GO with excellent water solubility, ease of functionalization and convenience in processing etc.^6^,^8^, making it the most popular precursor of graphene. Undoubtedly, it is of great significance to develop economical, eco-friendly and scalable routines to produce GO^3^,^9^,^10^.

As is well known, GO is synthesized dominantly via chemical oxidation of natural graphite even though there are a few reports on alternative electrochemical oxidation^11^,^12^. Back to 1859^13^, Brodie first synthesized graphite oxide by adding potassium chlorate to the slurry of graphite in fuming nitric acid. After about 40 years, Staudenmaier^14^ improved this method by replacing about two thirds of fuming HNO_3_ with concentrated H_2_SO_4_ and feeding the chlorate in batches. Based on these work, Hummers and Offeman^15^ developed an alternate oxidation method in 1958, often called Hummers method, in which NaNO_3_ and KMnO_4_ dissolved in concentrated H_2_SO_4_ was used to oxidize graphite into graphite oxide within a few hours. Thanks to the ease and short time of execution, Hummers’ method was widely adopted to afford GO^2^,^3^,^16^,^17^, but it still suffers from several flaws^18^,^19^,^20^,^21^,^22^,^23^,^24^, including toxic gas generation (NO_2_, N_2_O_4_), residual nitrate and low yield etc. To address these problems, various modification on Hummers’ method have been made in the past 20 years, and the main strategies can be summarized as follows: first^18^,^19^, removing NaNO_3_ directly from Hummers method with an improved workup; second^20^,^21^, adding a step of preoxidation before KMnO_4_ oxidation (in the absence of NaNO_3_); third^22^,^23^,^24^, increasing the amount of KMnO_4_ instead of NaNO_3_; fourth^25^,^26^,^27^, replacing KMnO_4_ with K_2_FeO_4_ while NaNO_3_ was removed. For example, in the report of Kovtyukhova *et al*.^20^, graphite was preoxidized by K_2_S_2_O_8_ and P_2_O_5_ before Hummers’ procedure was implemented. This work resulted in highly oxidized GO, but the whole process which contains solution transfer and material drying is rather time-consuming. By increasing the amount of both KMnO_4_ and concentrated H_2_SO_4_ (containing 1/9 H_3_PO_4_) instead of NaNO_3_, Marcano *et al*.^22^ found that the improved Hummers method leads to higher yield and the temperature can be easily controlled. Recently, Gao *et al*.^25^,^26^ reported a K_2_FeO_4_-based oxidation approach instead of KMnO_4_, and obtained single-layer GO at room temperature.

Despite the above progresses, two problems remain in various modified versions of Hummers method: (1) high consumption of the oxidants and intercalating agents was inevitable, (2) most of the synthesis routines proceed for a long time, both of which result in high cost and poor scalability in practical applications^19^,^28^. Therefore, there is a strong demand to develop an economical and efficient method for the synthesis of GO. In the present paper, we made a further improvement for NaNO_3_-free Hummers methods by partly replacing KMnO_4_ with K_2_FeO_4_ and reducing the amount of concentrated sulfuric acid. It shows that GO can be synthesized successfully with rather low auxiliary agents-to-graphite ratio within shorter reaction duration. The resultant GO was characterized by various instrumental methods, and an example of application was given for the GO derived graphene aerogel (GA) as supercapacitor electrodes. The present work provides a new possibility for the production of GO in an economical, eco-friendly and efficient way.

## Results

**Figure 1:**
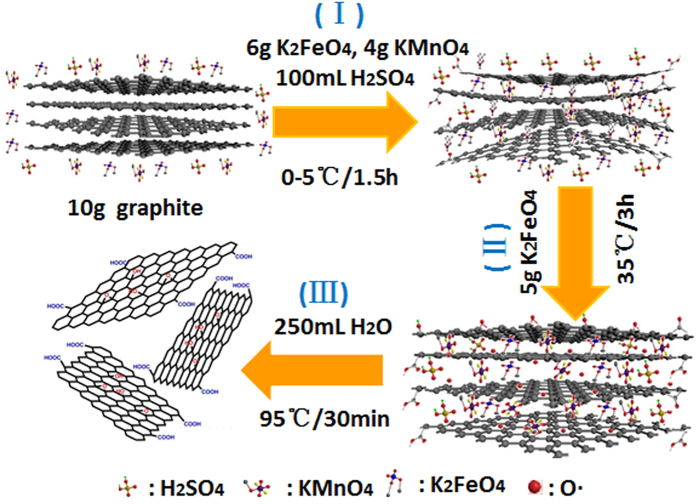
Illustration of the preparation of GO based on an newly improved Hummers method.

GO was prepared from natural flake graphite through one-pot synthesis based on new modified Hummers method. As illustrated in Fig. 1, the synthesis process consists of three critical steps^15^,^29^,^30^: (**I**) H_2_SO_4_ intercalation and boric acid stabilized^31^ K_2_FeO_4_/KMnO_4_ preoxidation at low temperature, during which H_2_SO_4_-graphite intercalation compounds (GIC)^32^ and then initial pristine graphite oxide (PGO)^9^ form, (**II**) deep oxidation with secondary feeding of KMnO_4_ at middle temperature, when GIC convert entirely into PGO by the diffusion-controlling oxidation^9^, and (**III**) hydrolysis and exfoliation of PGO into GO after the addition of H_2_O. The obtained GO was named GO2, while for comparison, another GO coined as GO1 was synthesized by Kovtyukhova improved method^20^. Further, GA was hydrothermally synthesized^33^,^34^ from GO1 and GO2, and named GA1 and GA2, respectively.

Table S1 illustrates the comparison of our GO and other GO synthesized by various methods with respect to the material ratio graphite:oxidant:solvent (Gr:Od:Sv, w/w/v), reaction time, C/O atomic ratio and the yield. It shows that in Hummers method, a typical material ratio of 1:3.5:23 and reaction time of about 2 hours are selected, which leads to low yield and toxic byproduct. To overcome these problems, high material ratio and long reaction time were employed in Kovtyukhova’s^20^ (Gr:Od:Sv = 1:6:50, 8 h) and Marcano’s^22^ (Gr:Od:Sv = 1:6:133, 12 h) work, and lower C/O ratio was achieved in the former work (1.98). In contrast, Chen’s^19^ and Gao’s^25^ routines reduced the reaction time, but the material ratio was still high. As compared to previous work, our strategy is much more economical with the least material consumption (Gr:Od:Sv = 1:1.5:10), short reaction time (5 h), and high yield (up to 84%). Our improvement works can be ascribed to following factors: (1) the intercalation and preoxidation of flake graphite is enhanced by using K_2_FeO_4_ as a stronger oxidant^25^,^35^ which partly replaces KMnO_4_ at extended low-temperature stage; (2) the secondary feeding of KMnO_4_ prevents it from fast decomposition and keeps the strong oxidability of concentrated sulfuric acid; (3) the reduced amount of sulfuric acid increases the concentration of graphite and oxidants, which improves the kinetics of the reaction and the utilization of the reactants.

**Figure 2:**
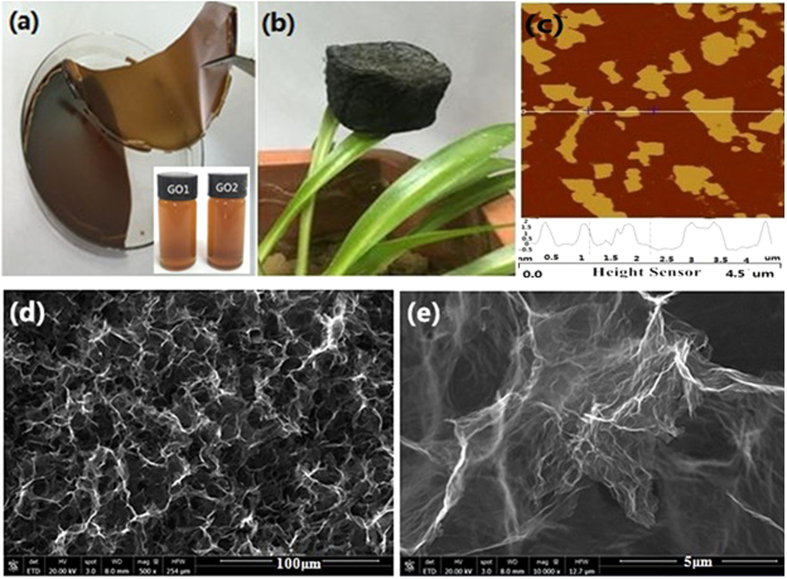
Photographs of (**a**) GO2 paper (the inset presenting GO1 and GO2 aqueous solutions) and (**b**) GA2, (**c**) AFM image of GO2, and (**d**,**e**) FESEM images of GA2.

Figure 2 demonstrates (a,b) the photographs, (c) AFM image and (d,e) FESEM images of several samples. As shown in the inset of Fig. 2a, both GO1 and GO2 aqueous solutions appear typical brownish, and there is little difference in color between them, which implies the GOs obtained from the two different methods are quite similar. A small amount of GO2 solution air-dried over a watch-glass gives a sheet of flexible GO2 paper (Fig. 2a), which is easily bent or tore. In contrast, GA2 exhibits a dark foam-like monolith (Fig. 2b), and it is so lightweight that can be supported over leaves of the pot plant. AFM images (Fig. 2c and Fig. S1) show the initial GO2 nanosheets are very irregular in morphology and have a lateral size of a few microns and a thickness of 1.5~2 nm, which corresponds to 2~3 layers of atoms. FESEM image (Fig. 2d) reveals GA2 is porous with abundant macropores of about 10~20 μm in size, meantime, a tremendous amount of reduced GO2 nanosheets self-assemble into three-dimensional conducting networks, as observed elsewhere^33^,^34^. These reduced GO2 nanosheets are so thin as to look transparent (Fig. 2e).

XRD patterns of the pristine graphite, GO1 and GO2 are given in Fig. S2. The strong peak at 2θ = 26.5° presents the (002) plane^7^,^9^ of pristine graphite with high crystallinity, while the weak peak located at 10~11° is an indicative of (001) plane of dispersed GO. The calculated interlayer spacing is 0.833 nm and 0.816 nm (see the inset) for GO1 and GO2, respectively, suggesting that both GO1 and GO2 were exfoliated thoroughly after the oxidation, and shared a similar interlayer spacing.

**Figure 3:**
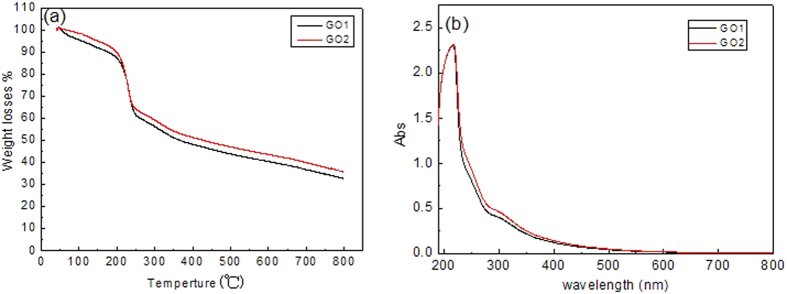
(**a**) TGA and (**b**) UV-vis absorbance of GO1 and GO2.

Figure 3 shows the TGA (a) and UV-vis spectra (b) of GO1 and GO2. In TGA (Fig. 3a), prominent weight loss occurs at about 200 °C, which is attributed to the pyrolysis of most oxygen-containing groups^18^,^19^, while the weight loss above 250 °C can be explained by the slow removal of the residual groups. The only difference is that weight loss of GO1 is slightly more than that of GO2, which implies more oxygen-containing groups formed in GO1. In UV-Vis spectra (Fig. 3b), the main peak at 230 nm and the shoulder peak at 300 nm stand for π-π* transitions of C=C bond from graphitic carbon of GO and n-π* transitions of C=O bond from oxidized carbon of GO^22^,^25^, respectively. Hence, GO1and GO2 exhibit similar characteristics in both TGA and UV-vis spectra, and indicate that they are similar in domain structures with similar oxygen groups.

**Figure 4:**
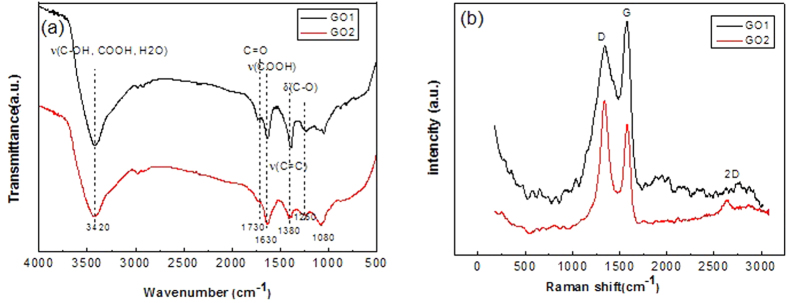
(**a**) FTIR and (**b**) RS of GO1 and GO2.

Figure 4 shows the typical FTIR (a) and RS (b) of GO1 and GO2. From the FTIR spectra, the same functional groups are identified for GO1 and GO2 as O–H stretching (3420 cm^−1^) and bending (1380 cm^−1^), C=O stretching (1730 cm^−1^), C=C stretching (1630 cm^−1^), C–O stretching (1260 cm^−1^) and C–O–C stretching (1080 cm^−1^) vibrations. The corresponding RS (Fig. 4b) were characterized by two typical peaks, G-band (1574 cm^−1^) and D-band (1360 cm^−1^). The G-band reflects the first order scattering of E_2g_ phonon of graphitic carbon, while D-band (1360 cm^−1^) relates to the formation of defects and disorder^25^,^36^, such as grain boundaries, hetero-atoms introduced in graphene planes, etc. In fact, GO sheet consists of two kinds of domains^37^: graphitic domains inherited from their parent graphite and highly oxidized domains due to oxidation. Therefore, D-band mainly records the information of GO distinct from graphite, especially the oxidation-induced defects and disorder, and the intensity ratio of D-band to G-band, I_D_/I_G_, indirectly reflects the oxidation degree^24^,^38^,^39^. Here the value of I_D_/I_G_ is 1.07 for GO1 and 0.94 for GO2, which indicates that abundant defects were introduced in GO during oxidation, and GO1 has a higher oxidation degree than GO2. A similar phenomenon was reported by Kim group^36^.

**Figure 5:**
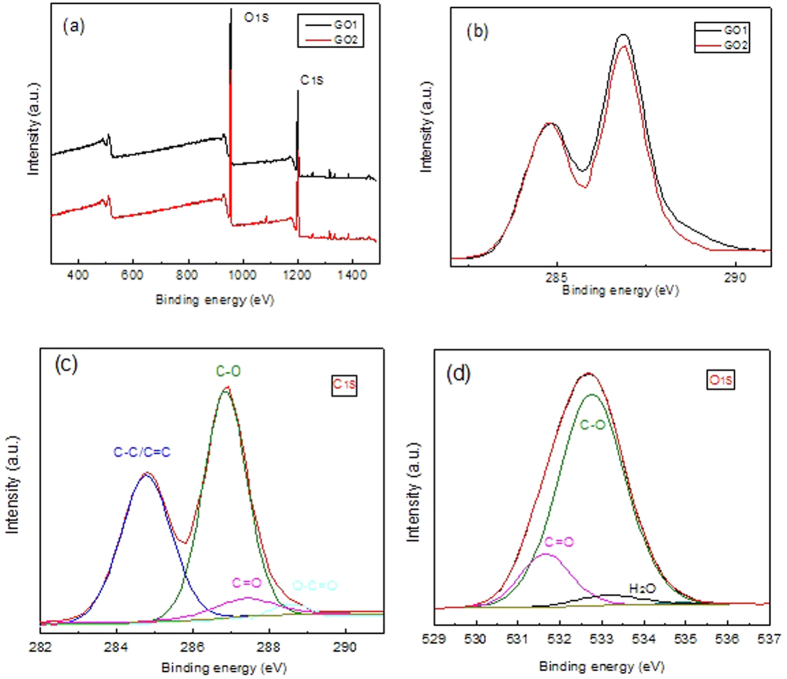
XPS of samples (**a**) survey spectrum of GO1 and GO2, (**b**) C1s of GO1 and GO2 normalized to C=C peak, (**c**) deconvoluted C1s of GO2 and (**d**) deconvoluted O1s of GO2.

The chemical states of GO1 and GO2 were investigated by XPS (Fig. 5). Figure 5a shows GO1 and GO2 have very similar survey spectra where C and O coexist as main elements. GO1 and GO2 were further compared with respect to C1s spectra normalized to C=C peak (284.6 eV) in Fig. 5b. It is clear that GO1 has a higher intensity of right peak correlated with oxidized carbon in C1s. In addition, quantitative analysis reveals that the C to O ratio is 1.98 for GO1 and 2.12 for GO2, respectively. The two points above arrive at a consistent conclusion that GO was successfully synthesized^10^, and GO2 has a lower oxidation degree than GO1^22^, supporting the results of TGA and RS. To resolve the bond components, C1s and O1s spectra of GO2 were deconvoluted in Fig. 5c,d, respectively. Figure 5c shows four types of covalently bonded carbon existing in GO2 as sp^2^ carbon (C-C/C=C, 284.6 eV), epoxy/hydroxyl groups (C–O, 286.6 eV), carbonyl groups (C=O, 287.8 eV) and carboxyl groups (O–C=O, 289.5 eV), respectively. Meanwhile, the oxygen in GO2 (Fig. 5d) can be resolved in the form of C=O bond (531.3 eV), C-O bond (532.4 eV) and possibly adsorbed H_2_O (533.2 eV). These results agree with the reported data of GO^19^,^22^,^25^.

**Figure 6:**
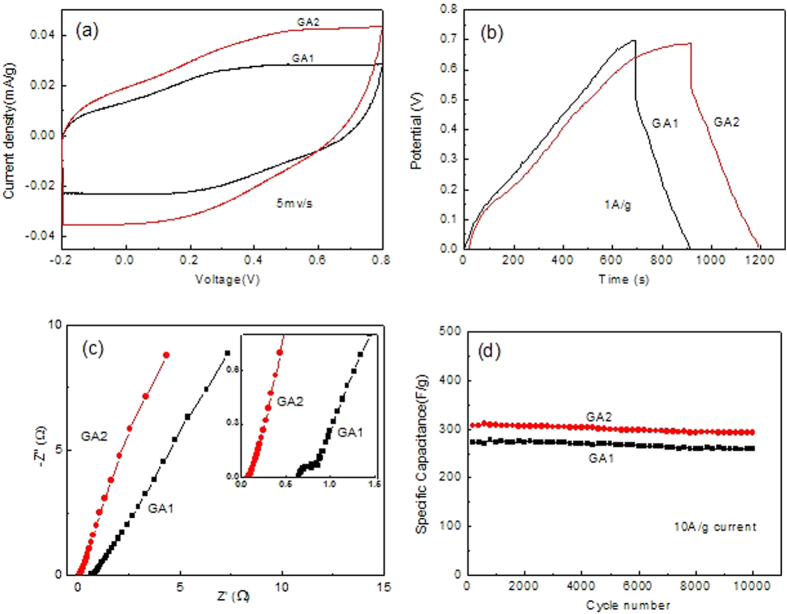
Comparison of electrochemical properties of GA1 and GA2 (**a**) CV, (**b**) GCD, (**c**) EIS and (**d**) cycle stability curves.

As an example of GO application, GA was tested as the free-standing supercapacitor electrode. As shown in Fig. 6, the electrochemical properties of GA were characterized by CV, GCD, EIS and cycle stability curves, respectively. The nearly rectangular CV curves (Fig. 6a) obtained at a scan rate of 5 mV/s reveal mostly the electrical-double-layer (EDL) capacitance characteristic of GA^33^,^34^, and GA2 has a bigger area of CV loop and specific capacitance as compared to GA1. The nearly linear and symmetric GCD curves (Fig. 6b) are another characterization of EDL capacitance. In the curves, the initial vertical section of discharge curve presents the voltage drop correlated with inner resistance (IR) of electrodes, so GA2 has smaller IR than GA1. This can be further confirmed by the EIS (Fig. 6c), in which the intersection (standing for the IR) of GA2 curve is clearly ahead of that of GA1. Finally, we evaluated the circle stability of GA in Fig. 6d by plotting specific capacitance (calculated from GCD curves^7^,^34^) vs charge/discharge number, where large current density of 10A/g was applied. It shows that upon circling for 10000 times, the capacitance of both GA1 and GA2 keeps almost constant. This excellent stability can be ascribed to the robust three-dimensional conducting networks within GA, which provide direct and stable pathway of electron transport required for EDL capacitance. In addition, higher capacitance and lower IR obtained in GA2 than those of GA1 can be explained by the fact that GO2 suffered from weaker oxidation and lower structural defect than GO1. Full comparison of electrochemical properties between GA1 and GA2 was summarized in Table S2. The good electrochemical properties indicate that GA2 is a promising candidate for EDL supercapacitor electrodes.

## Discussion

In summary, to synthesize GO economically and efficiently, we made an attempt to improve the existing NaNO_3_-free Hummers methods based on a one-pot routine by three main points: first, partly replacing KMnO_4_ with K_2_FeO_4_ of higher oxidability at low temperature to enhance the intercalation and preoxidation of graphite; second, two-step feeding of KMnO_4_ elevates the utilization of oxidants; third, reduced amount of concentrated H_2_SO_4_ increases the concentration of graphite and oxidants, and improves the kinetics of oxidation process. As compared to those protocols reported, our routine has high competitiveness in material consumption, both for the oxidants and the intercalating agents, meanwhile, the reaction can be completed within a shorter time. We demonstrated that with the ingredient ratio of graphite: oxidant: intercalating agent = 1:1.5:10 (w/w/v), graphite can be efficiently converted to GO within 5 hours. The improved Hummers method can be used to synthesize GO and its derivatives in an economical, eco-friendly, and large-scale way.

## Methods

### Materials

Natural flake graphite (325 meshes) was purchased from Qingdao Meilikun Co. Ltd., China, K_2_FeO_4_(AR) was provided by Hubei CSW Chemistry Co., Ltd., China. All other regents were received from Aladdin Industrial Corporation and used without any further purification.

### Synthesis of GO

Typically, flake graphite (10 g), KMnO_4_ (6 g) and K_2_FeO_4_ (4 g) as the oxidants, and boric acid (0.01 g) as a stabilizer were first dispersed in 100 mL of concentrated sulfuric acid in a vessel and stirred for 1.5 h at less than 5 °C. After the addition of another KMnO_4_ (5 g), the vessel was transferred into a water bath at about 35 °C and stirred for another 3 h to complete the deep oxidation. Next, as 250 mL of deionized water was slowly added, the temperature was adjusted to 95 °C and held for 15 minutes, when the diluted suspension turned brown, indicating the hydrolysis and absolute exfoliation of intercalated graphite oxide. Finally, this brown suspension was further treated with 12 mL H_2_O_2_ (30%) to reduce the residual oxidants and intermediates to soluble sulfate, then centrifuged at 10000 rpm for 20 min to remove the residual graphite, and washed with 1 mol/L HCl and deionized water repeatedly, producing the terminal GO (designated GO2). For comparison, another GO (designated GO1) was synthesized following Kovtyukhova improved Hummers method^20^, except two of little modification: (1) the ingredients were adjusted slightly, as shown in Table S1; (2) both preoxidation and oxidation time was set at 4 hours.

### Synthesis of GA

GA was prepared from GO according to hydrothermal method^33^,^34^. Typically, 25 mL of 2 mg/ml GO1 (or GO2) aqueous solution obtained above was sealed in a Teflon lined stainless-steel autoclave, and hydrothermally reduced at 180 °C for 12 h. After the autoclave was cooled and discharged, the reduced GO1 (or GO2) was obtained and subsequently freeze-dried for 24 h at −55 °C, which gives a porous graphene monolith, coined as GA1 (or GA2).

### Preparation of GA electrode

GA slice (diameter ~1 cm, thickness~1 mm) was cut from GA bulk, then mechanically pressed over a Ni foam under a pressure of 10 MPa, acting as GA electrode.

### Characterization of materials

Optical absorption spectra of GO solution were taken from an ultraviolet visible spectrophotometer (UV-vis, Shimadzu UV-3600). Chemical structure of GO was analyzed using Fourier transformation infrared spectroscopy (FTIR, Bruker IFS66V), Raman spectroscopy (RS, Renishaw) and X-ray photoelectron spectroscopy (XPS, Kratos Axis Supra) with Al-Ka radiation). The weight loss of samples was measured by thermal gravimetric analyzer (TGA, STA 449 C TG-DSC) with a heating rate of 10 °C/min under Ar gas flow of 50 mL/min. The phases were identified by X-ray diffractometer (XRD, PANalytical X’Pert PRO) with Cu-Ka radiation. The morphology of samples was characterized using field-emission scanning electron microscopy (FESEM, JEOL JSM-6701F) and atomic force microscopy (AFM, Bruker, Dimension edge).

### Electrochemical tests

Cyclic voltammetry(CV), galvanostatic charge/discharge(GCD) and electrochemical impedance spectroscopy (EIS) were measured based on a three-electrode cell system: the GA electrode as working electrode, a Pt plate as counter electrode and a calomel electrode as reference electrode, respectively, which uses 1 mol/L of H_2_SO_4_ as electrolyte. The CV and EIS were acquired using an electrochemical workstation (CHI 660E, Shanghai CHI, China), while GCD performance was taken by a Battery Test System (Land 2001, Wuhan Kingnuo, China).

## Additional Information

**How to cite this article**: Yu, H. *et al*. High-efficient Synthesis of Graphene Oxide Based on Improved Hummers Method. *Sci. Rep*. **6**, 36143; doi: 10.1038/srep36143 (2016).

**Publisher’s note:** Springer Nature remains neutral with regard to jurisdictional claims in published maps and institutional affiliations.

## Supplementary Material

Supplementary Information
