# Supplementary material for: High-efficient Synthesis of Graphene Oxide Based on Improved Hummers Method
Source: Sci Rep. 2016 Nov 3;6:36143. doi: 10.1038/srep36143 (PMC5093679; doi:10.1038/srep36143)
Supplement: Supplementary Information [file srep36143-s1.pdf]

Supplementary

High-efficient Synthesis of Graphene Oxide Based on Improved Hummers Method

Huitao Yu, Bangwen Zhang*, Chaoke Bulin, Ruihong Li, Ruiguang Xing

College of Materials and Metallurgy, Inner Mongolia University of Science and Technology, Baotou 014010, China

*Corresponding author

Bangwen Zhang Prof.: bangwenz@126.com

Table S1 Comparison of synthesis condition and product properties of GO through various methods

Method	graphite	oxidant	solvents	Gr:Od:Sv (w/w/v)	reaction time	C/O atomic ratio of GO	yield of GO
Brodie method [11]	-	-	fNA:cSA=1:4	-	3-4d	2.16	low
Staudenmaier method[12]	1g	30g	40mL fNA + 20mL cSA	1:3:60	1-10d	2.89	low
Hummers method [13]	100g	300g $KMnO_4$ +50g $NaNO_3$	2300mL cSA	1:3.5:23	2h	2.1-2.9	40%[23]
Chen method[14]	3g	9g $KMnO_4$	70mL cSA	1:3:23	2h	2.36	-
Kovtyukhova method[16]	10g	40g$KMnO_4$,10g$K_2S_2O_8$,10gP_2O_5	500mL cSA	1:6:50	>8h	1.98	92%
Marcano method[18]	3g	18g $KMnO_4$	360mLcSA +40mLcPA	1:6:133	>12h	-	77%
Gao method [21-22]	10g	60g $KMnO_4$	400mL cSA	1:6:40	1~6h	2.2	-
Present method	10g	9g $KMnO_4$ 6g $KFeO_4$	100mL cSA	1:1.5:10	5h	2.12	84%

Note: cSA(concentrated sulfuric acid), cPA(concentrated phosphoric acid), fNA(fuming nitric acid), Gr(graphite), Od(Oxidant), Sv(solvents or acid).

Table S2 Comparison of electrochemical properties between GA1 and GA2

Samples	Specific capacitance ($F\cdot g^{-1}$)	GCD		EIS	Cycle Stability Curves
		Charging time (s)	Discharge time (s)	(Internal Resistance)	(Loss degree)
GA1	273	690	219	0.6	5.1%
GA2	307	910	280	0.1	4.8%

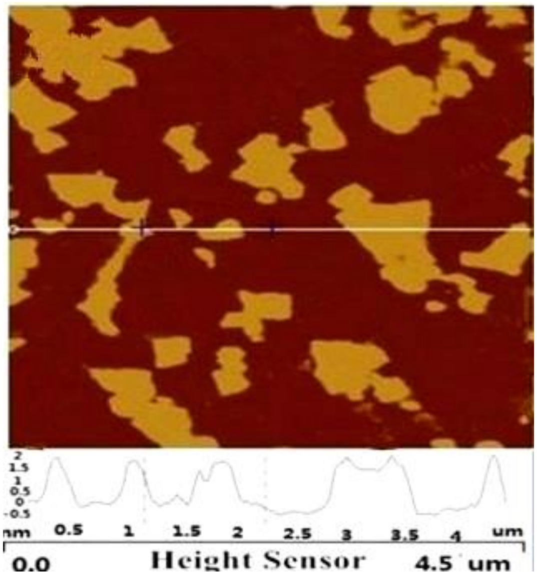

Fig.S1 AFM image of GO2

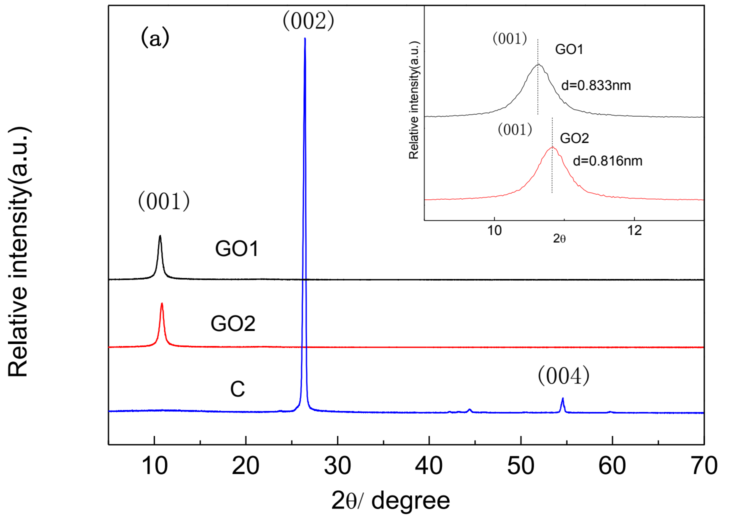

Fig.S2 XRD patterns of Graphite, GO1 and GO2
